# Epigenetic and accelerated age in captive olive baboons (*Papio anubis*), and relationships with walking speed and fine motor performance

**DOI:** 10.18632/aging.206223

**Published:** 2025-03-18

**Authors:** Sarah J. Neal, Shannon Whitney, Soojin V. Yi, Joe H. Simmons

**Affiliations:** 1 The University of Texas MD Anderson Cancer Center, Michale E. Keeling Center for Comparative Medicine and Research, National Center for Chimpanzee Care, TX 78602, USA; 2 Texas State University, Department of Biology Supple Science Building, TX 78666, USA; 3 Department of Ecology and Evolution and Marine Biology, Department of Molecular, Cellular and Developmental Biology, Neuroscience Research Institute, University of California, Santa Barbara, CA 93106, USA

**Keywords:** epigenetics, DNA methylation, baboons, behavioral indicators, accelerating aging

## Abstract

Epigenetic age, estimated by DNA methylation across the genome, reflects biological age. Accelerated age (i.e., an older methylation age than expected given chronological age) is an accepted aging biomarker in humans, showing robust associations with deleterious health outcomes, longevity, and mortality. However, data regarding age acceleration in nonhuman primates (NHPs), and relationships between NHP epigenetic age and behavioral indicators of aging, such as walking speed and fine motor performance, are sparse. We measured DNA methylation of 140 captive olive baboons (*Papio anubis*) (84% female, 3-20 years-old), estimated their epigenetic ages, and classified them as showing age acceleration or deceleration. We found that epigenetic age was strongly correlated with chronological age, and that approximately 27% of the sample showed age acceleration and 28% showed age deceleration. We subsequently examined relationships between epigenetic and accelerated age and walking speed (N=129) and fine motor performance (N=39). Older animals showed slower speeds and poorer motor performance. However, the difference between the epigenetic age and chronological age, referred to as delta age, was not a consistent predictor of walking speed or fine motor performance. These data highlight the need for further examination of age acceleration across NHP species, and the ways that age acceleration may (not) be related to indicators of aging in NHP models.

## INTRODUCTION

DNA methylation is a widely studied epigenetic mechanism involved in several key processes including regulation of gene expression, genomic imprinting, development, disease, to name a few [1–3]. In particular, recent studies have utilized epigenetic age, a measure of biological age that is based on levels of DNA methylation across the genome, as a robust biomarker of aging [4–6]. Levels of DNA methylation increase or decrease with chronological age [3, 6–11] as well as with a variety of lifestyle factors [12], longevity [13], mortality [5, 14], obesity [15], cancer [16], and various age-related diseases, including cognitive decline and neurodegeneration [16, 17]. The difference between an individual’s chronological age and their epigenetic age is termed “delta age.” When an individual’s delta age is positive (i.e., their epigenetic age is greater or older than their chronological age), that person is said to exhibit age-acceleration, indicating that they are aging more quickly than they should, based on their chronological age. Like epigenetic age, age acceleration has been associated with a plethora of deleterious outcomes, poor prognoses, morbidity, and mortality [5, 10, 17–23]. External factors, including obesity, tobacco use, early life adversity, lifetime stress, and traumatic events, among others, also contribute to accelerated aging [24–29].

Although many studies have examined relationships between health outcomes and epigenetic and accelerated age in humans, few studies have investigated these associations in nonhuman primates (NHPs). Given that NHPs serve as important models of human aging and aging-related diseases, these investigations are important to elucidate mechanisms and develop interventions of such diseases and disorders. Although epigenetic clocks (i.e., models using DNA methylation to estimate chronological age) have been developed for rhesus macaques [30], baboons [31, 32], chimpanzees [33, 34], and marmosets [35], few studies have examined the discrepancy between chronological and epigenetic age in NHP species (i.e., accelerated or decelerated age). To our knowledge, only a handful studies have examined associations between accelerated age and health outcomes in NHPs, each of which are in baboons, and show that accelerated age is linked to stress. One study found age acceleration in high status male baboons, potentially indicating that high social status is a stressful and costly position to maintain, resulting in accelerated age [31]. Two additional studies found that intrauterine growth restriction and fetal undernutrition was linked to accelerated aging in the brain and cardiovascular systems in baboons [36, 37]. Furthermore, some NHPs are nursery-reared, consisting of maternal separation and human-rearing. Nursery-reared individuals tend to exhibit increased abnormal and stress-related behaviors, altered immune function, and poorer overall health [38–40]. Given this early-life adversity, we would perhaps expect accelerated aging in these nursery-reared individuals compared to their mother-reared counterparts, especially given the fact that lifetime stress exposure in humans has been associated with accelerated aging [29]. However, no studies have examined this hypothesis. Given the overall lack of data on this topic, it is unknown whether NHPs exhibit age acceleration in the same ways as humans, and if so, whether age acceleration in NHPs is associated with deleterious outcomes and aging indicators.

Walking speed is a useful behavioral indicator of aging in humans and NHPs, with slower walking speeds associated with older age [41, 42], cognitive decline, deleterious cardiovascular events, dementia, as well as overall morbidity and mortality [43–49]. The relationship between slow walking speeds and older age has been observed in several NHP species [41, 42, 50–52]. NHPs also show relationships between slower walking speeds, depression, and overall physical decline [31, 41, 42, 50, 53]. Given that it is simple, inexpensive, unobtrusive, and sensitive to age, walking speed is a popular measure in NHP aging studies [50].

**Figure 1:**
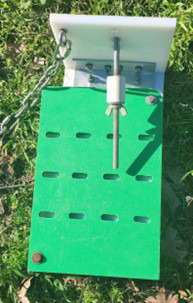
The fine motor task apparatus.

In humans, fine motor function, including speed, dexterity, and strength, decreases with age [54–56]. As such, clinical assessments used for aging-related diseases often include measures of fine motor function [55]. Like humans, NHPs also show age-related decrements in motor ability. For example, robust age-related decreases in fine motor performance have been found in gorillas [57], marmosets [58], and rhesus macaques [59–61]. Although baboons show decreases in gross motor performance with age [62], no data exist regarding potential age-related changes in fine motor performance in this species. One commonly used test in NHPs is a variation of the Brinkman board, in which the subject removes a small object, such as a piece of food, from small holes in a board (e.g., Figure 1) [63, 64]. This assessment measures the level of precision grip, which requires the complex coordination of the thumb and index finger, along with the muscles in the arm, hand, and shoulder [64]. Given that age-related decrements in fine motor skill have been shown across various species of NHP, fine motor performance could be considered a behavioral indicator of aging, similar to walking speed.

Given that walking speed and fine motor performance are considered behavioral indicators of aging, it follows that they should be correlated with DNA methylation-based estimates of age (i.e., epigenetic age), and that individuals with accelerated age may exhibit altered walking speed and motor performance outcomes. In the current study, we first aimed to generate epigenetic ages of baboons using DNA methylation estimates and subsequently compare these epigenetic ages to chronological ages. We also aimed to examine the discrepancy between chronological and epigenetic age in order to define the level of age acceleration and deceleration within our sample based on sex, rearing, and age group. Our second aim was to explore the relationship between epigenetic age and behavioral indicators of aging, including walking speed and fine motor performance. We were specifically interested in comparing the strength of chronological, epigenetic, and accelerated age in predicting these behavioral indicators.

## RESULTS

### Baboons show age acceleration and deceleration

Blood samples for DNA methylation analyses were collected from 140 captive olive baboons (*Papio anubis*, 118 female, 22 male; 86 nursery-reared, 52 mother-reared, and 2 with an unknown rearing history) housed at the Michale E. Keeling Center for Comparative Medicine and Research of The University of Texas MD Anderson Cancer Center in Bastrop, Texas. At the time of sample collection, animals were in good overall health, as indicated by bloodwork results within normal limits and only minor trauma noted during exams. Genomic DNA was extracted and used to construct reduced representation bisulfite sequencing (RRBS) libraries, which were sequenced with an Illumina HiSeq3000 (Materials and Methods). RRBS reads were mapped to the baboon papAnu4 reference genome to quantify DNA methylation at base level resolution. To avoid errors due to SNPs, we excluded positions that are known to harbor polymorphisms from a survey of 100 baboons (Materials and Methods). After these steps, we constructed a DNA epigenetic clock (Materials and Methods). The resulting baboon epigenetic clock accurately predicted chronological age using the levels of DNA methylation across 153 CpG sites.

**Figure 2:**
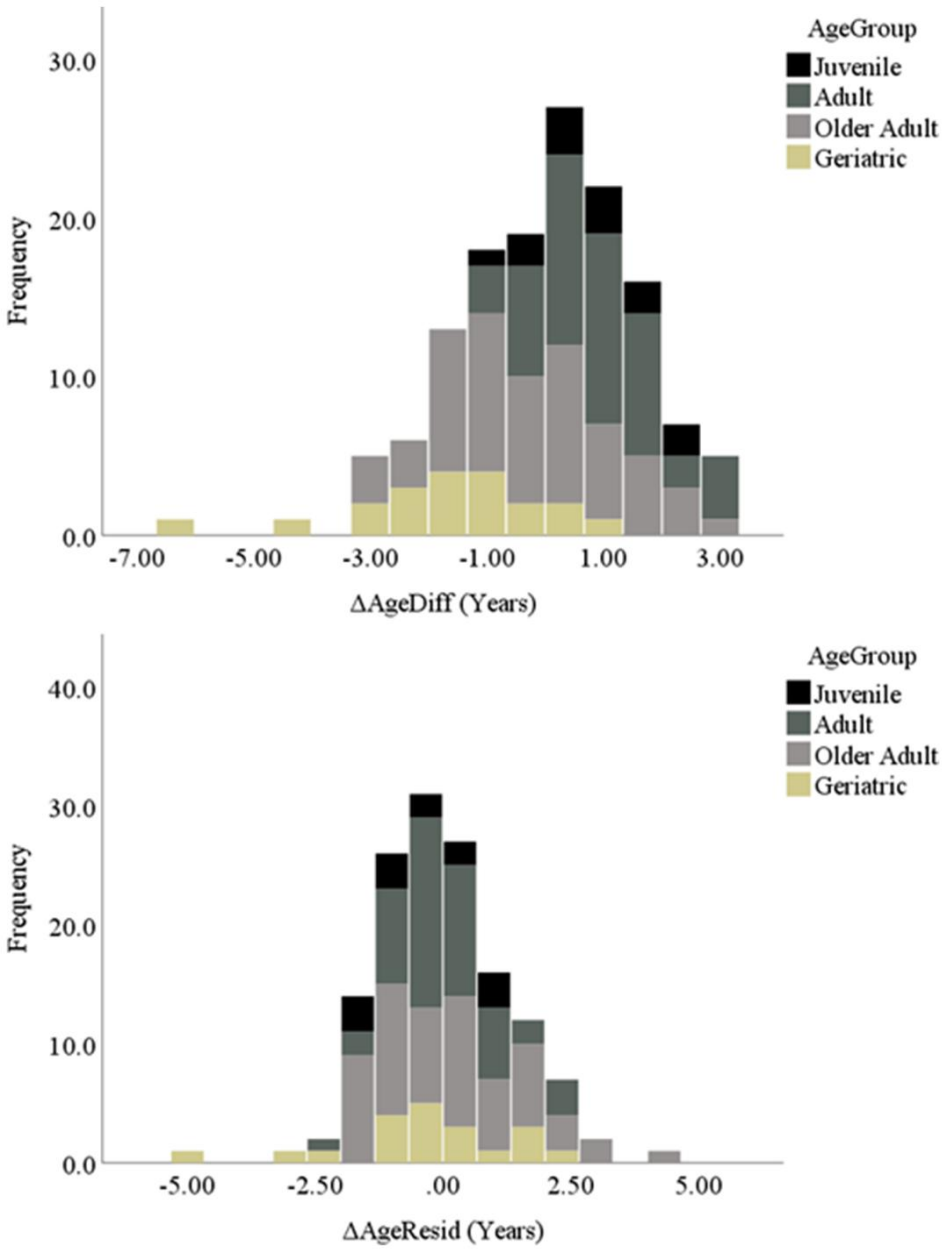
Histograms of both delta age variables separated by age group.

We then calculated two delta age (ΔAge) measures, representing the difference between epigenetic age and chronological age for each baboon. First, we calculated delta age representing the difference between epigenetic and chronological age for each baboon, termed ΔAgeDiff (i.e., “delta age difference”). Second, we calculated delta age as the regression residual between chronological and epigenetic age, obtained by regressing epigenetic age onto chronological age and saving the unstandardized residual, termed ΔAgeResid (i.e., “delta age residual”). Using these two variables, baboons were then categorized as showing age deceleration, acceleration, or a relative match according to the parameters described in detail in the Methods. These two measures yielded slightly different categorizations of age-accelerated and -decelerated baboons (Table 1). Additionally, as shown in Figure 2, the two measures showed slightly different categorizations across age group (juvenile, young adult, older adult, and geriatric [65]). According to these categorizations of age acceleration and deceleration, approximately 19% (using ΔAgeResid) to 27% (using ΔAgeDiff) of our sample exhibited age acceleration (i.e., epigenetic age one or more years greater than chronological age, approximately equivalent to greater than 3 years of age acceleration in humans [62, 66]). Another approximately 21% (ΔAgeResid) to 28% (ΔAgeDiff) of the sample showed age deceleration, and approximately 45% (ΔAgeDiff) to 58% (ΔAgeResid) showed a relative match between their epigenetic and chronological ages.

**Table 1:** Descriptive statistics of age accelerated and decelerated baboons.

		**-1**	**0**	**1**
		**Age deceleration**	**Relative match**	**Age acceleration**
ΔAgeDiff	n	39	63	38
	Mean Delta	-1.94 years	0.11 years	1.75 years
	Range	-1.07 to -6.47 years	-0.97 to 0.97 years	1.03 to 3.29 years
ΔAgeResid	n	30	83	27
	Mean Delta	-1.63	-0.02	1.89
	Range	-1.63 to -4.94	-0.99 to 1.00 years	1.01 to 4.06 years

**Figure 3:**
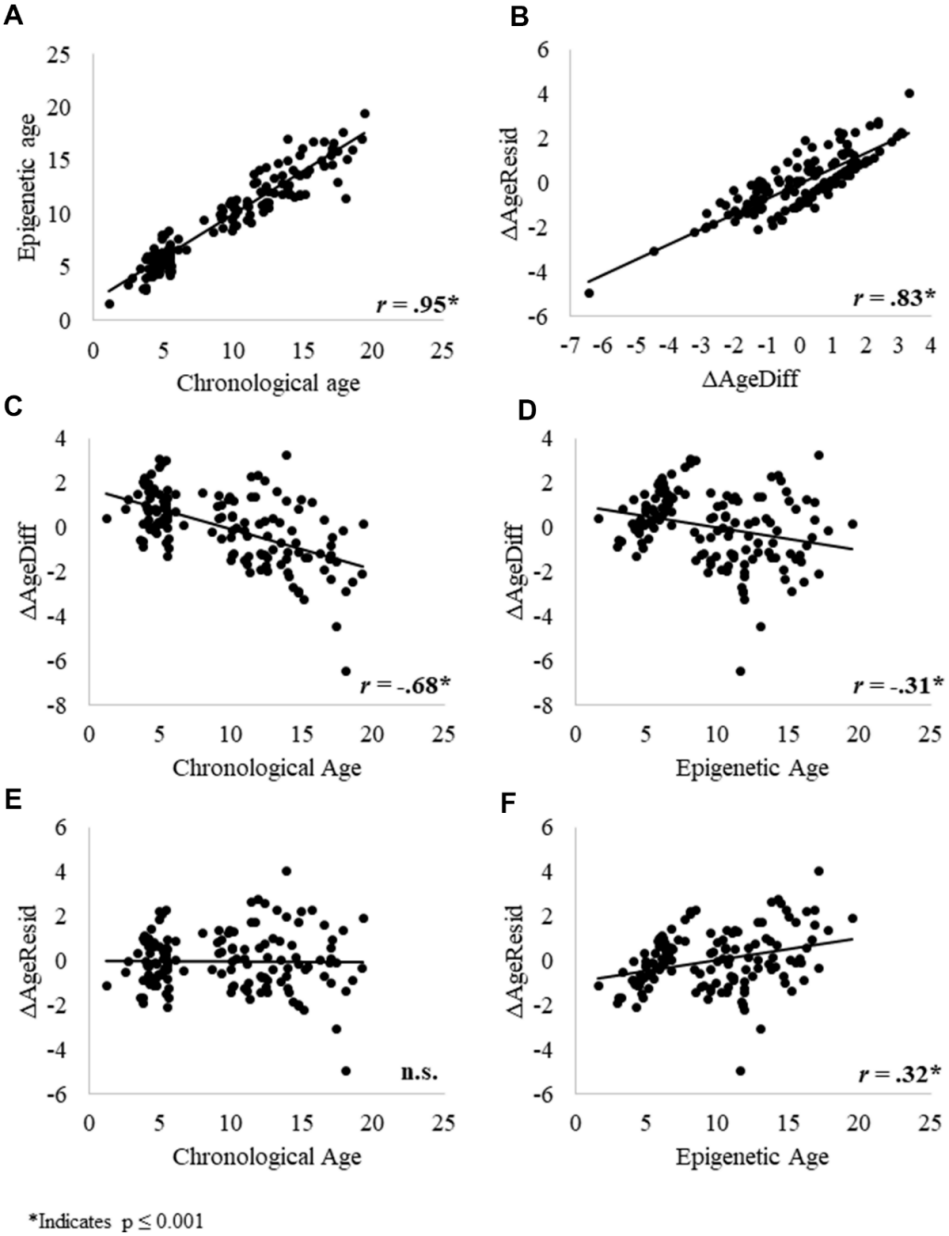
Relationships between (**A**) epigenetic age and chronological age; (**B**) the two delta age measures: ΔAgeDiff and ΔAgeResid; (**C**) ΔAgeDiff and chronological age; (**D**) ΔAgeDiff and epigenetic age; (**E**) ΔAgeResid and chronological age; and (**F**) ΔAgeResid and epigenetic age. Given that these relationships were similar across sexes, we combined results for males and females. * Indicates p ≤ 0.001.

We then examined relationships between the epigenetic, chronological, and delta age variables. Consistent with previous research, chronological age significantly predicted epigenetic age (Figure 3A). ΔAgeResid and ΔAgeDiff were significantly, positively correlated (Figure 3B), indicating that these two variables measure correlated aspects of aging. ΔAgeDiff was significantly negatively correlated with both chronological and epigenetic age (Figure 3C and 3D), whereas ΔAgeResid was not correlated with chronological age (Figure 3E) but was significantly positively correlated with epigenetic age (Figure 3F). At face value, the negative correlations between ΔAgeDiff and chronological and epigenetic age seem to indicate that the epigenetic clock tends to underestimate age for older individuals.

### Mixed evidence that younger baboons show age acceleration, while older baboons show age deceleration

**Figure 4:**
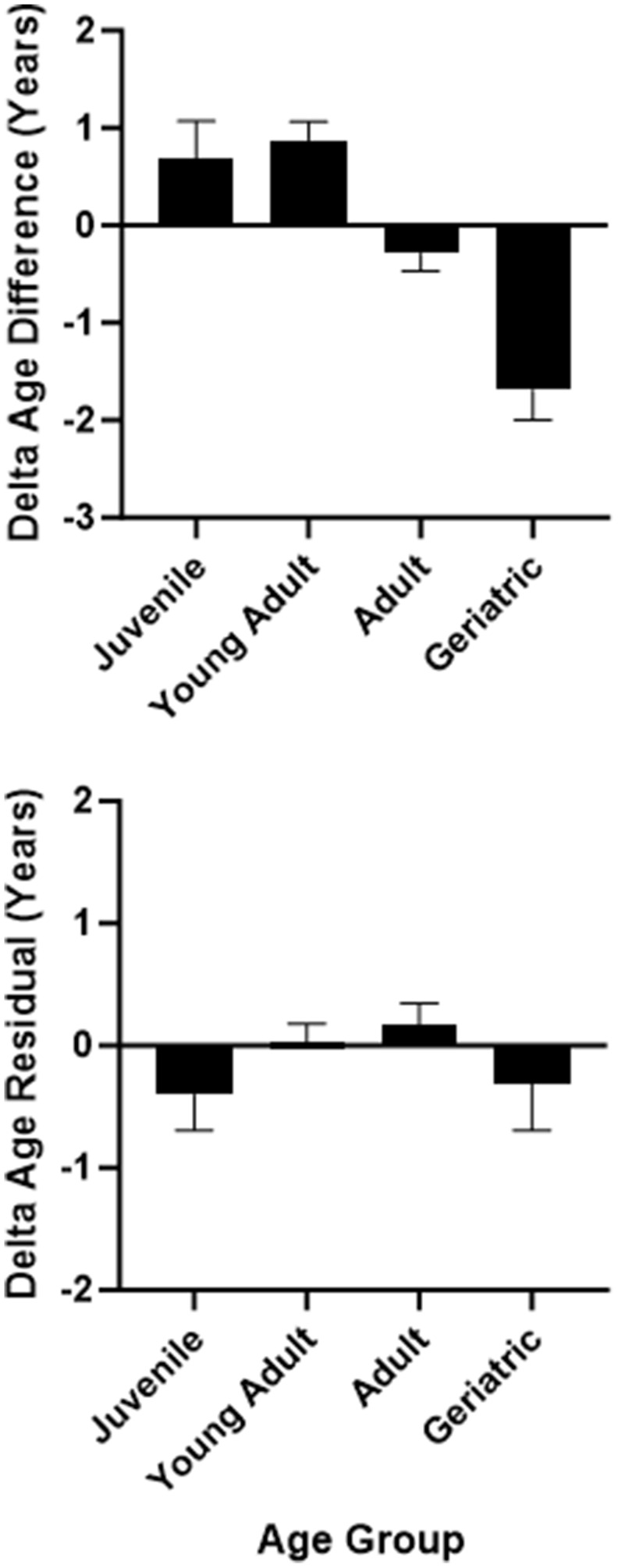
Delta age (difference measure in top panel and residual measure in bottom panel) as a function of age group.

A univariate ANOVA with sex, rearing, and age group as between-subjects factors showed that ΔAgeDiff values were not significantly different as a function of sex or rearing (p>0.10), but differed significantly across age groups, F(3,124) = 13.93, p < 0.001 (Figure 4). Geriatric baboons had the lowest ΔAgeDiff, corresponding to age deceleration, followed by older adult, younger adult, and juvenile baboons. Bonferroni post-hoc tests showed that all age groups were significantly different from each other (p < 0.001) except between juveniles and young adults (p > 0.90) and juveniles and older adults (p > 0.10). It should be noted that the juvenile age category was primarily composed of mother-reared females. Therefore, we selected only adults within the dataset (young adult and older adult age categories with equal representation across sexes and rearing statuses) and repeated the above ANOVA. The results were replicated, F(1,97) = 17.01, p < 0.001. However, when repeating the analysis with the second delta age variable (ΔAgeResid), age acceleration was not related to sex, rearing, or age group (p>0.35). As such, while one delta age measure showed that older baboons tended to show age deceleration, the other measure showed no such relationship.

### Chronological and epigenetic age predict aging behavioral indicators equally well

Descriptive statistics showed that walking speed in our sample ranged from 0.40 meters/second (m/s) to 1.01 m/s (mean = 0.63 m/s, SEM = 0.019), and fine motor performance ranged from 0.18 raisins retrieved per second (r/s) to 0.61 r/s (mean = 0.42 r/s, SEM = 0.022). Walking speed and fine motor performance were not correlated (Pearson’s correlation r(39) = .08, p = 0.62).

**Figure 5:**
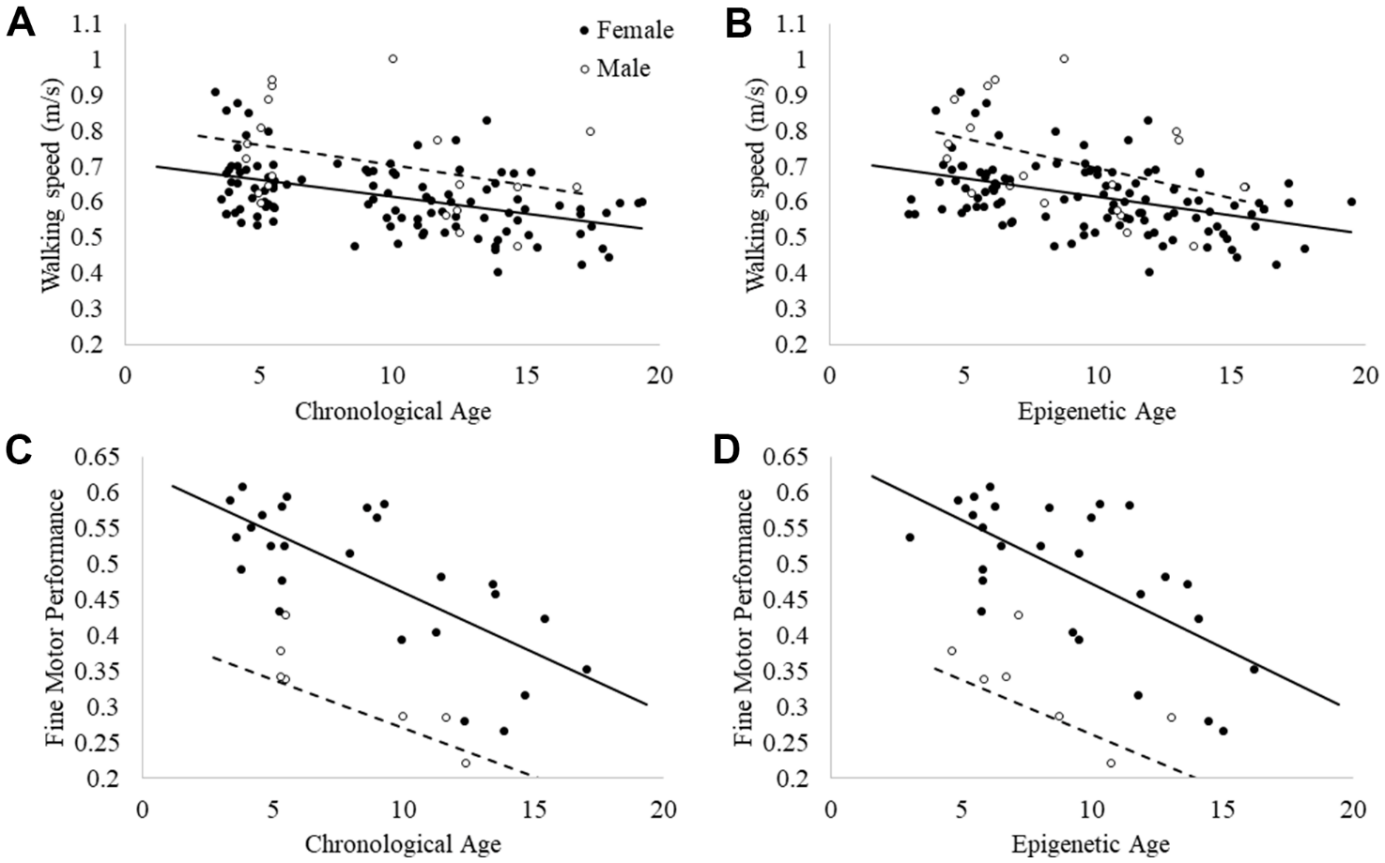
Relationships between (**A**) walking speed and chronological age; (**B**) walking speed and epigenetic age; (**C**) fine motor task performance and chronological age; and (**D**) fine motor task performance and epigenetic age.

Consistent with previous research [41, 42, 50], chronological age significantly predicted walking speed, F(3,123) = 15.87, p = 0.0001, *R^2^_adj_* =.261, beta = -0.009, p = 0.0001 (Figure 5A). For one additional year of life, baboon walking speed decreased an average of 0.009 meters per second (approximately 21 inches per minute). Sex was also a significant predictor of walking speed, with males walking faster than females (beta = .103, p = 0.001). Rearing was not a significant predictor in the model (p>0.50).

Epigenetic age also significantly predicted walking speed, F(3,123) = 15.46, p = 0.0001, *R^2^_adj_* =.26, beta = -0.010, p = 0.0001 (Figure 5B). Similar to the model with chronological age, for each additional year of epigenetic age, baboon walking speed decreased by 0.010 meters per second (approximately 23 inches per minute). Sex was also a significant predictor of walking speed, with males walking faster than females, beta = 0.098, p = 0.001. Rearing was not a significant predictor in the model (p>0.5).

Chronological age also significantly predicted fine motor performance, F(3,34) = 34.73, p = 0.0001, *R^2^_adj_* =.73, beta = -0.014, p = 0.0001 (see Figure 5C), with poorer performance in older baboons. Sex was also a significant predictor in the model, with females showing faster performance than males: beta = -.19, p = 0.001, although rearing was not (p>0.40). Epigenetic age also significantly predicted fine motor performance, F(3,34) = 29.18, p = 0.0001, *R^2^_adj_* =.70, beta = -0.016, p = 0.0001 (Figure 5D). Sex was also a significant predictor in the model, with females showing faster performance than males: beta = -.205, p = 0.001, but rearing was not (p>0.40). As such, chronological and epigenetic age performed similarly well in predicting both behavioral indicators of aging.

### Mixed evidence that accelerated and decelerated age affect walking speed and fine motor performance

We then explored the effects of age acceleration, sex, and rearing on walking speed. Using ΔAgeDiff, we found that walking speed was significantly different as a function of delta age. Baboons showing age deceleration (M = 0.64 m/s, SEM = 0.02) and those showing age acceleration (M = 0.64 m/s, SEM = 0.02) walked significantly slower than those showing a relative match between their chronological and epigenetic age (M = .72 m/s, SEM = 0.02), F(2, 115) = 3.84, p = 0.03. However, this effect of age acceleration was not found using the second delta age variable (ΔAgeResid) (p> 0.20). Sex was a significant predictor in both models: Females (M = 0.62, SEM = 0.011) walked significantly slower than males (M = 0.72, SEM = 0.025), p<0.001. Walking speed was not significantly different as a function of rearing in either model (p > .15). We could not assess interaction effects given the small number of males in the sample.

Lastly, we explored the effects of age acceleration, sex, and rearing on fine motor performance. Fine motor performance was significantly different as a function of ΔAgeDiff: baboons showing age deceleration (M = 0.31, SEM = 0.03) retrieved fewer raisins per second than those showing age acceleration (M = 0.42, SEM = 0.03) and those showing a relative match between their chronological and epigenetic age (M = .43, SEM = 0.03), F(2, 27) = 3.28, p = 0.05. Although there was not a significant difference in fine motor performance as a function of age acceleration using the ΔAgeResid measure, the raw means showed similar effects to the model using the ΔAgeDiff measure: baboons showing age deceleration (M = 0.31, SEM = 0.04) retrieved fewer raisins per second than those showing age acceleration (M = 0.41, SEM = 0.04) and those showing a relative match between their chronological and epigenetic age (M = .44, SEM = 0.02), p = 0.29. In both models, sex was a significant predictor, with females (M = 0.46, SEM = 0.012) having retrieved significantly more raisins per second than males (M = 0.28, SEM = 0.027), p = 0.002. Fine motor performance was not significantly different as a function of rearing in either model (p > 0.60), and we could not assess interaction effects given the small number of males in the sample.

## DISCUSSION

Our first aim was to investigate epigenetic ages of the current baboon cohort. We decided to use an unbiased genome-wide reduced representation bisulfite sequencing (RRBS) to develop a DNA methylation clock. Because epigenetic clock studies use reduced sampling to identify small numbers of predictive CpGs from the genome, clocks developed from other studies do not necessarily have the same predictive power in a specific cohort. In addition, determining DNA methylation values of a few hundred specific clock CpGs is experimentally more prohibitive than an unbiased sampling of CpGs. For example, 448 (78%) of clock sites from a previous clock [31] were found in our RRBS data. The predicted ages from these sites showed a greater dispersion and thus a reduced fit compared to the clock developed from our cohort (*R*^2^ = 0.65). This could be also influenced by genetic polymorphism present in a different subspecies of baboon used in the previous study. Our clock sites would thus increase the potential CpG positions that can be used to predict epigenetic ages from baboon derived samples/tissues.

These are some of the first data to classify baboons as age-accelerated or -decelerated [31], and examine relationships between epigenetic age, age acceleration, and behavioral indicators of aging. Age acceleration is strongly correlated with health and aging outcomes in humans [5, 13, 14, 16, 17], but only one study, to our knowledge, has examined accelerated aging in baboons [31]. Therefore, we aimed to investigate the relationship between age acceleration and two behavioral indicators of health and aging commonly used in NHPs that are also associated with age in humans: walking speed and fine motor performance [43–49, 54–56]. We opted to utilize two measures of delta age to examine whether the difference between chronological and epigenetic age was related to sex, rearing, and age group. The first (ΔAgeDiff) was a basic and intuitive measure representing the raw numerical difference between each baboon’s epigenetic and chronological age. Although not often used in the literature, this measure provides an easy-to-interpret indication of each individual baboon’s level of age acceleration or deceleration. The second measure (ΔAgeResid) represents the magnitude of the difference between epigenetic and chronological age compared to the average difference (i.e., the residual difference between chronological age and the regression line). While this measure is often used in the literature, it can be prone to bias given that the regression line is an estimation of the linear relationship between chronological and epigenetic age based on the data input [67]. Although the two delta age measures were strongly correlated, they yielded slightly different results regarding 1) categorizations of baboons as age-accelerated or -decelerated; 2) relationships with sex, rearing, and age group; and 3) associations with walking speed and fine motor performance. We believe that the differences in the resulting associations between these two measures and aging indicators highlights how different conclusions may be reached based on which measure is utilized.

First, the two delta age measures categorized individual baboons slightly differently across age acceleration and deceleration. As shown in the histograms (Figure 2), the distribution of delta ages of the ΔAgeResid measure showed a tighter grouping of values around 0 (i.e., a higher number of baboons showing a relative match between chronological and epigenetic age) and fewer extreme values than the ΔAgeDiff measure. As such, it seems that ΔAgeResid was a slightly more conservative measure of age acceleration and deceleration compared to ΔAgeDiff, which may be due to its calculation of the residual from the average (i.e., the regression line) rather than the difference between chronological and epigenetic age. Regardless, the two measures indicate that approximately one quarter of baboons in our sample show age acceleration and another quarter show age deceleration. Furthermore, some baboons exhibited up to 5 years of age acceleration or deceleration, approximately equivalent to 18 years in humans [62, 66]. Therefore, both measures indicate substantial variation between chronological and epigenetic age in some baboon individuals.

Second, the two delta age measures yielded slightly different associations with sex, rearing, and age group, which may be due to the smaller sample size of accelerated and decelerated individuals in the more conservative ΔAgeResid measure. While neither measure showed an association with sex nor rearing, ΔAgeDiff showed an association with age group whereas ΔAgeResid did not. The ΔAgeDiff measure showed that juveniles, adults, and older adults tended to show a relative match between their epigenetic and chronological age (i.e., less than one-year discrepancy), whereas geriatric baboons showed a significant amount of age deceleration. Indeed, baboons in the geriatric age category showed an average of almost 2 years age deceleration according to this measure, approximately equivalent to 6 years for an age-decelerated human.

In humans, one study found that epigenetic age showed a steady linear increase with chronological age, but only up to very old age, at which point the linear increase between epigenetic and chronological age began to slow [5]. In this older age category, epigenetic age began to predict chronological age, on average, 1.4 years slower, indicating age deceleration. Therefore, while epigenetic age accurately predicted chronological age in the young and middle-aged groups, it began to underestimate age in the oldest age groups. This is consistent with additional data showing that the Horvath clock underestimated chronological age by 4 years, on average [14, 68]. The authors suggest that the slower rate of change of epigenetic age in comparison to chronological age may be due to selective survival: old individuals with age deceleration are living to old age precisely because of that age deceleration [5]. The authors hypothesized that individuals with age acceleration had the highest mortality, leaving the surviving population comprised of individuals with age deceleration, and this hypothesis was supported by a survival analysis [5]. This phenomenon of selective survival has also been posited in the relationship between chimpanzee neutrophil to lymphocyte ratio (NLR) and mortality. In humans, NLR is robustly predictive of mortality and increases with age [69]. In contrast, chimpanzee NLR is significantly lower in the oldest individuals, a finding that was then replicated in baboons [70]. The authors posited that chimpanzees with higher NLRs died at younger ages, whereas those with lower NLRs survived into old age [71]. It is possible that the age deceleration of geriatric baboons found in the present study reflects a similar phenomenon. However, recall that the ΔAgeResid measure showed no such difference across age group. As mentioned previously this may be due to the smaller sample size of age-accelerated and -decelerated baboons derived from this measure’s calculation. Given the mixed evidence of this association, more data are needed to further clarify this relationship.

It is interesting that neither delta age measure differed as a function of rearing in the current sample given that nursery-rearing in NHPs is often used as a model for early-life adversity [38–40, 72], and, in humans, early life adversity is associated with age acceleration [29]. It has previously been noted that nursery-reared baboons tend to show less detrimental outcomes as a consequence of nursery-rearing compared to other NHP species (e.g., macaques) [38]. It is possible that the stress of such rearing practices (or lack thereof) is not affecting epigenetic modifications to the genome. Alternatively, given that age and rearing were confounded in our sample (i.e., juvenile baboons were primarily mother-reared), it is possible that rearing has an effect but that it was masked by the effect of age group in our analyses. However, we do not believe this to be the case since rearing remained a non-significant predictor of delta age when we re-ran the analyses with only adults (equally representing rearing and sex) in the dataset. This result should be re-evaluated in a larger sample of baboons, and accelerated aging as a function of rearing should be examined in other NHPs. Additionally, our results showed no effects of sex on age acceleration using either delta age measure, which is inconsistent with a recent systematic review showing that male sex was predictive of age acceleration in humans [73]. Like the results with rearing and delta age, the relationship between delta age and sex should be examined in a larger sample with additional males.

Third, we examined associations between epigenetic age, delta age, and behavioral indicators of aging: walking speed and fine motor performance. Consistent with previous research, our results showed a negative relationship between both behavioral indicators and chronological age in baboons [41, 42, 50]. To our knowledge, ours is the first study to demonstrate that walking speed and fine motor performance are also negatively associated with epigenetic age, although this result may be unsurprising given the strong correlation between chronological and epigenetic age. We were particularly interested in determining whether epigenetic age, as a measure of biological age, was a better predictor of walking speed and fine motor performance compared to chronological age. The results showed only minor differences in the strength of chronological and epigenetic age to predict walking speed and fine motor performance. Based on the overall effect sizes (*R^2^* statistics in the regression models), chronological and epigenetic age seemed to predict both behavioral indicators relatively equally well. One of the criteria for the American Federation for Aging Research (AFAR) for aging indicators is that the biomarker should predict the rate of aging, and be a better predictor of lifespan than chronological age [74, 75]. Given that epigenetic age is thought to reflect biological age, the significant association between epigenetic age and both walking speed and fine motor performance further demonstrates the utility of these measures as behavioral indicators of aging. However, more research is needed to elucidate whether walking speed and fine motor performance can increase prediction of lifespan over and above chronological age alone.

We also found inconsistent results across the two delta age measures in predicting walking speed and fine motor performance. Using the ΔAgeDiff measure, we found that baboons with accelerated age and decelerated age both showed slower walking speeds compared to those showing a relative match between their chronological and epigenetic age. Additionally, the ΔAgeDiff measure showed that baboons with age deceleration performed worse on the fine motor task. However, it should be noted that this result is likely confounded by age group, since baboons with age deceleration were more likely to be geriatric. However, neither of these results were replicated using the ΔAgeResid measure. Age acceleration is associated with a host of deleterious outcomes in humans [5, 19, 21], as well as stressful circumstances in NHPs [31] (although it is worth noting that longitudinal studies have found that accelerated age in humans did not predict walking speed [68] but was associated with taking fewer steps and decreased grip strength [76]). As such, we expected walking speed and fine motor performance to be negatively associated with accelerated age, such that individuals with age acceleration would show slower walking speeds and worse performance on the fine motor task. Although one delta age measure seems to provide some preliminary evidence supporting an association between behavioral aging indicators and deviations between chronological and epigenetic age, the inconsistency of the associations precludes any definitive conclusions [68, 76].

The current study was limited by the age, sex, and rearing distribution of the sample. As mentioned previously, rearing, age, and sex were confounded, such that the majority of geriatric baboons were nursery-reared females, and we had very few males in the sample, particularly in older age categories. Although studies demonstrate that rearing in baboons may result in only minimal differences in health parameters (e.g., immune parameters and body weight, but no differences in reproduction, behavior, or wounding [77–80]), this is in contrast to studies in rhesus macaques showing widespread effects of rearing on health, behavior, immunology, and welfare [40, 81, 82]. Additionally, sample size across age groups (juvenile: 13, young adult: 49, older adult: 58, and geriatric: 20) was unequal. Therefore, the effects of such covariates, especially rearing, must be re-evaluated in future studies with a more balanced cohort. Nevertheless, we re-ran analyses with a subset of baboons with equal representation of sex and rearing, and replicated the lack of rearing and sex effects from the larger analysis. Regardless, given that human studies show that male sex was predictive of age acceleration, additional studies with larger sample sizes, including a larger sample of males and greater equality across rearing and age groups, are needed to add to the data on accelerated aging and relationships with demographic variables and aging outcomes in NHPs.

Molecular tools to assess DNA methylation are rapidly evolving, and different methods can result in slightly different epigenetic clocks as well as outcomes regarding relationships with mortality, morbidity, and health consequences [83]. It has been argued that certain first-generation clocks are more prone to error due to noise during detection of CpG sites, and second- and third-generation clocks have been developed to combat such issues [67, 83]. Given the rapidly evolving technology, future studies should aim to evaluate differences in DNA methylation-based age estimates across clocks for NHPs, and how this may impact the characterization of age acceleration and deceleration. Another necessary improvement for future studies is to examine epigenetic clocks in a cell-type resolution. Given that blood samples consist of several cell types and that different cell types may change their epigenetic profiles differently, accounting for cell type heterogeneity could improve our understanding of epigenetic ages.

In conclusion, these data demonstrate that baboons exhibit varying degrees of differences between their chronological and epigenetic ages (i.e., their delta age), allowing characterization of baboons as age-accelerated or decelerated. However, more data are needed to determine the functional consequences of age acceleration in baboons, as there was mixed evidence of delta age affecting behavioral indicators of aging, including walking speed and fine motor performance. As such, additional exploration of the baboon as a model for the effects of aging is warranted. Further evaluations of delta age in the context of demographics, health, aging indicators, and mortality are needed to elucidate the validity and utility of age acceleration as an aging biomarker in NHPs.

## MATERIALS AND METHODS

### Subjects

Baboons were housed across 18 separate social groups ranging in size from 3 to 37 baboons per group, and ranged in age from 1.17-19.33 years (mean age = 9.54 years), with 20 geriatric baboons (i.e., ≥15 years of age). For some analyses, baboons were divided into four age groups: 1) juvenile (4 years old or younger, n = 13); 2) young adult (5-9 years of age, n = 49); 3) older adult (10-14 years of age, n = 58); 4) geriatric (15 years or older, n = 20).

Baboons were housed in corrals or Primadomes™ with indoor-outdoor access. Both Primadomes™ and corrals included various physical environmental enrichment items, including, but not limited to, climbing structures with platforms, culvert sections, various sizes of plastic balls, 55-gallon barrels, and fire hose rope/swings. Baboons were also provided with daily foraging opportunities and enrichment devices.

### DNA methylation

We used genome wide methylation analysis to construct epigenetic clocks and determine epigenetic ages. Blood samples were collected during routine biannual physical exams in the spring of 2021. DNA was extracted from blood specimens using QIAGEN DNeasy Blood and Tissue kits (Qiagen) following manufacturer protocols in a Level 2 Biological Safety Cabinet. DNA was brought to a standard concentration of ~70 ng/μL either by dilution with nuclease-free water or concentration using Millipore Microcon Centrifugal Filter Devices. Bisulfite conversion of DNA and methylation assays were performed at the University of Texas MD Anderson Cancer Center’s Epigenetics Profiling Center (Sequencing and Genomics Core). We used reduced-representation bisulfite sequencing (RRBS) data to estimate methylation across the genome. The RRBS library was constructed using NuGEN Library preparation kit (NuGEN) according to the manufacturer protocol. The libraries were loaded onto an Illumina HiSeq3000 system for sequencing using 57 bp single-end reads.

To estimate DNA methylation, adapter trimming and quality control were done using TrimGalore v.0.4.1 with a default setting. The libraries from the NuGEN kit use a 6-base barcode with an additional 6 random bases which can be used for determining duplicate reads. We removed the additional adaptor sequences added by the diversity adaptors using custom python script provided by NuGEN Technologies (https://github.com/nugentechnologies/NuMetRRBS). The sequencing reads were mapped to the baboon papAnu4 reference genome using Bismark v 0.14. Duplicated reads were removed using the deduplicate module built in the Bismark software program. Because genetic polymorphisms of thymine at CpG sites are not distinguishable from bisulfite-converted cytosines, we removed polymorphic CpGs from downstream analyses to avoid incorrect methylation calls due to the technical limitation of distinguishing bisulfite converted thymine from unmethylated cytosine. Genetic variants collected from 100 baboons were downloaded from [84].

To estimate epigenetic age, we removed CpGs with a mean methylation level either less than 0.1 or greater than 0.9 in order to retain informative CpG sites. Also, we removed CpGs with a mean depth of coverage less than 5. We excluded CpG sites with missing data in any individuals. DNA methylation clock for baboons was built using elastic net regression. We followed the methods in Anderson et al. (2021) with minor modifications to predict epigenetic ages. Briefly, using normalized levels of DNA methylation at 373,185 candidate clock CpG sites as our predictor values and the chronological age as the observed outcome, linear models were constructed. The R package *glmnet* [85] was used to perform elastic net regression analysis. We used the alpha parameter of 0.5. We set the regularization parameter lambda to the value which minimizes the mean squared error during cross validation. To estimate the methylation age of individual samples, we want to clarify that we performed a leave-one-out cross-validation without including any samples in the training dataset to avoid overfitting. The trained model was then used to predict the left-out test sample’s age.

### Walking speed

Walking speeds were collected on 129 baboons (109 female, 18 male; 78 mother-reared, 49 nursery-reared, 4 with an unknown rearing history and excluded from analyses; 10 juvenile, 45 adult, 55 older adult, 17 geriatric). Landmarks within corrals and enclosures were measured. A single researcher used a stopwatch to opportunistically measure the time to walk between recorded landmarks. Only bouts of walking past both markers were recorded, each bout had to be separated by 5 seconds of sitting or standing still. Bouts of walking toward food and/or those involving social interaction were not recorded to account for underlying motivation. A minimum of 10 bouts of walking were recorded per individual. Walking speed was calculated as distance (meters) / time (seconds). The average walking speed per individual was used for statistical analysis. All observations were conducted outdoors, and between the hours of 8 am – 5 pm. Although walking speed has been found to be unaffected by temperature and humidity [41], data collection was conducted while temperatures were between 10 &deg;C and 37 &deg;C. Data were collected between November 2021 and August 2022.

### Fine motor task performance

Fine motor task performance was collected on 39 of the 129 baboons (27 female, 11 male; 20 mother-reared, 18 nursery-reared, 1 with an unknown rearing history and excluded from analyses; 4 juvenile, 16 adult, 14 older adult, 4 geriatric). To measure fine motor ability, we used a variation of the Brinkman board, as used previously with NHPs [57, 60, 64]. This board uses small crevices to encourage the animal to pinch for a reward. This task was selected because it required little training for the animals, and was able to be mounted to the enclosure. A total of 12 oval crevices were made in an HPDE panel (see Figure 1), and one raisin was pushed into each oval. The device was hung on the outside of the enclosure, requiring that the baboon reach outside of the enclosure bars to obtain the raisins. A research assistant measured the amount of time it took for an animal to collect all raisins. An individual failed a trial if they took longer than 3 minutes to obtain all raisins. Each animal was presented with the board for two sessions, each consisting of four trials. We recorded the number of raisins picked and the time elapsed, with the dependent variable expressed as raisins per second. Therefore, higher scores on this measure represent faster fine motor performance.

### Data analysis

### Delta age

We calculated two measures of delta age. First, we calculated the difference between predicted age and chronological age and termed this “ΔAgeDiff,” representing age acceleration or deceleration using the difference between epigenetic and chronological age (epigenetic age – chronological age = delta age). The second measure of delta age was calculated by regressing epigenetic age onto chronological age and saving the unstandardized residuals [86], representing the magnitude of difference between epigenetic and chronological age compared to the average (represented by the regression line). We termed this “ΔAgeResid,” representing age acceleration or deceleration using the residuals from the regression. Therefore, for both measures, positive values represented epigenetic ages older than chronological age (i.e., age acceleration) and negative values represented epigenetic ages younger than chronological age (i.e., age deceleration).

We then created categories of age acceleration and deceleration using both ΔAgeDiff and ΔAgeResid. Baboons whose epigenetic age was within 1 year (-1 year to +1 year) of their chronological age were assigned a 0, representing a relative match between their epigenetic and chronological ages. Baboons that had a younger epigenetic than chronological age by at least 1 year (delta ages of -1.00 years or lower) were assigned a value of -1, representing age deceleration. Finally, baboons that had an older epigenetic age than chronological age by at least 1 year (+1.00 year or higher) were assigned a value of +1, representing age acceleration (see Table 1).

To characterize age acceleration and deceleration across our sample, we used univariate ANOVAs to explore differences in ΔAgeDiff and ΔAgeResid as a function of sex, rearing, and age group (as described above: juvenile, adult, older adult, geriatric). Due to the low number of males in the sample, as well as the high number of nursery-reared females in the older age categories, we could not assess interaction effects. As such, only main effects are reported. We performed these analyses with both delta age variables.

### Relationships between epigenetic age, delta age, walking speed, and fine motor performance

We used linear regressions to examine the effects of chronological and epigenetic age on walking speed and fine motor performance. We were particularly interested in whether chronological or epigenetic age is a better predictor of these behavioral indicators of aging, as evaluated by the p-values and *R^2^* of the models, with a higher *R^2^* indicating a higher proportion of the variance of the dependent variables explained by the predictors. As such, sex and rearing were entered on the first block of each equation, walking speed and fine motor performance served as the outcome variables, and each age variable served as the predictor in each separate regression.

We then used univariate ANOVAs to examine differences in walking speed and fine motor performance as a function of accelerated age, sex, and rearing. Walking speed and fine motor performance served as the outcome variables, with sex, rearing, and the categorized age acceleration variables (ΔAgeDiff and ΔAgeResid) (-1: age deceleration; 0: relative match; +1: age acceleration) as the between-groups factors. All analyses were performed in IBM SPSS v.26. Data are available from the corresponding author upon reasonable request.
